# Utility of the CT Scan in Diagnosing Midgut Volvulus in Patients with Chronic Abdominal Pain

**DOI:** 10.1155/2017/1079192

**Published:** 2017-01-15

**Authors:** Ehsan Shahverdi, Mehdi Morshedi, Maryam Allahverdi Khani, Mohammad Baradaran Jamili, Fatemeh Shafizadeh Barmi

**Affiliations:** ^1^Student Research Committee, Baqiyatallah University of Medical Sciences, Tehran, Iran; ^2^Blood Transfusion Research Center, High Institute for Research and Education in Transfusion Medicine, Tehran, Iran; ^3^Department of Surgery, Baqiyatallah University of Medical Sciences, Tehran, Iran; ^4^Department of Medicine, Najafabad Branch, Islamic Azad University, Najafabad, Iran; ^5^Department of Radiology, Boo Ali Hospital, Tehran Medical Sciences Branch, Islamic Azad University, Tehran, Iran; ^6^Department of Microbiology, Tehran Medical Sciences Branch, Islamic Azad University, Tehran, Iran

## Abstract

Symptomatic intestinal malrotation first presenting in the adults is rare. Midgut volvulus is the most common complication of malrotation in the adults. Because of more differential diagnosis, Computed Tomography (CT) scan can play an important role in the evaluation of patients with this abnormality. The whirl pattern around the superior mesenteric artery found on CT scan in patients with midgut volvulus is pathognomonic and diagnostic. We describe a case of intestinal malrotation complicated by midgut volvulus in an adult patient. The preoperative CT findings were pathognomonic.

## 1. Introduction

A volvulus is a subtype of malrotation, in which a loop of bowel twisted about a focal point along the mesentery attached to the intestinal tract, which may result in a bowel obstruction [1].

Midgut volvulus is more frequent in children and young adults but rarely presented in adults. In the old age, it may be a different obstruction and most importantly compromising blood flow to and from the bowel wall threatening intestinal viability [2, 3]. Malabsorption and steatorrhea may be due to partial occlusion veins and lymphatics. The peptic ulcer occurs in 20% of patients, probably because the current recession is in the atrium duodenum [2]. Diagnosis of intestinal malrotation in adults rarely occurs. Computed Tomography (CT) scan can play a major role in the evaluation of intestinal malrotation.

Herein, we describe a 52-year-old female with diagnosis of midgut volvulus with chronic abdominal pain and bowel obstruction.

*Ethical Considerations*. The patient was asked to sign an informed consent form. All the terms of the Helsinki Declaration were considered, and the personal information remained anonymous.

## 2. Case Report

A 52-year-old female was admitted to Baqiyatallah Hospital, Tehran, Iran, in 2015 with a history of seven-month generalized abdominal pain slightly more in the epigastrium, accompanied by nausea and vomiting. She had a history of intermittent abdominal pain, recurrent nausea and vomiting, inability in gas passing, and constipation with at least ten times of hospitalization during the past three years. Her past medical history was significant for asthma, tubal ligation and umbilical hernia surgery, cholecystectomy, diagnostic laparoscopy, and simultaneous appendectomy.

At the admission, vital signs were the pulse 85 beats per minute; temperature 37.2°C, and blood pressure 110/75 mmHg. Abdominal examination revealed generalized tenderness with preference in the epigastric and right abdomen without rebound and guarding, and it was distended slightly. Her rectal examination was normal.

## 3. Laboratory Tests and Images

White blood cell (WBC) count was 9900/mm^3^ with 24% lymphocytes; hemoglobin was 16.1 g/dL, and hematocrit was 46.6% (normal: 35.9–44.6). All biochemistry tests were in normal range. Endoscopic findings showed bile reflux, erythematous gastritis, and stomach body ulcer. Abdominal sonography study was normal.

**Figure 1:**
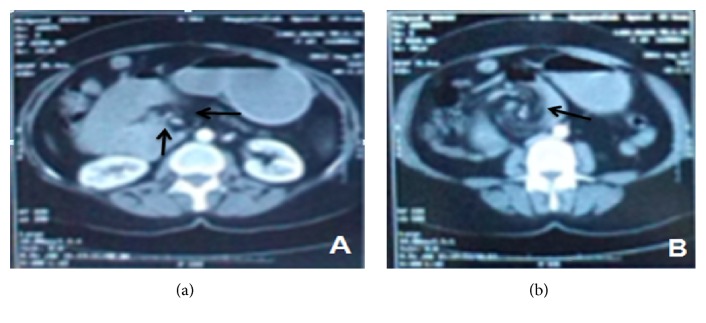
(a-b) CT scans obtained at the time of hospital admission. (a) shows collaterals of superior mesenteric veins surrounding the superior mesenteric artery. In (b) bowel is twisted around central superior mesenteric artery.

**Figure 2:**
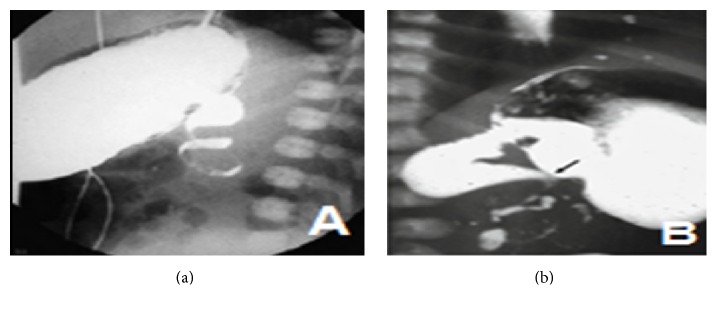
(a) Upper GI series shows the “corkscrew sign” in a frontal view. (b) Upper GI series shows malrotation with midgut volvulus. An incomplete duodenal obstruction and dilation of the first and second portions are seen, as is the “corkscrew sign.”

Abdominal CT scan showed torsion around the narrow mesenteric stalk (Figures 1(a) and 1(b)). Abnormal findings included positioning of the duodenojejunal flexure to the right of the spine, obstruction of the duodenum, and “beak” appearance of the obstructed proximal jejunum (Figures 2(a) and 2(b)).

**Figure 3:**
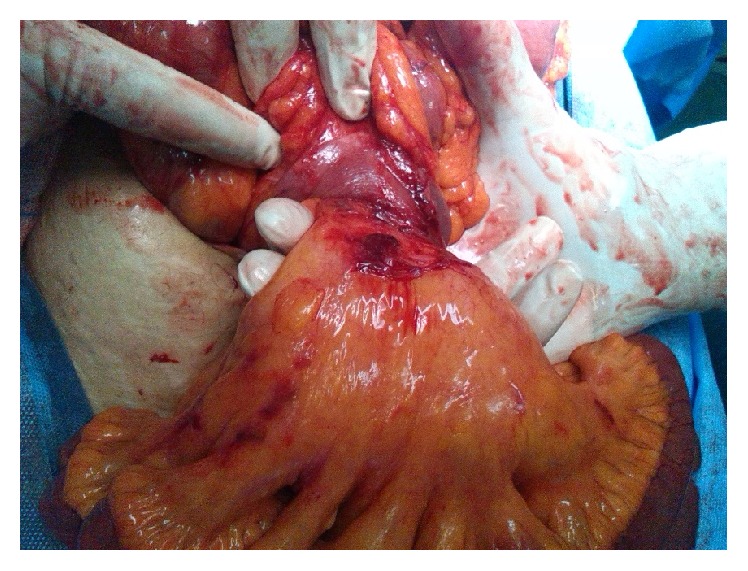
Intestinal malrotation with midgut volvulus.

With the impression of intestinal volvulus, the patient was brought to the operating room, and the patient underwent surgical correction by Ladd's procedure. She remains asymptomatic 5 months after surgery (Figure 3).

## 4. Discussion

The volvulus is defined as the twisting of the intestines around mesentery due to intestinal obstruction [3]. The incidence of midgut volvulus in Africa and Asia is more than Western countries (24–60 per 100,000 population versus 1.7–5.7 per 100,000 population) [2, 3].

Pain caused by midgut volvulus usually presented as an umbilical or epigastric pain, frequently postprandial and often associated with a feeling of fullness, nausea, and vomiting.

Midgut volvulus symptoms include chronic abdominal pain, nausea, bloating or flatulence, the appearance of blood in the stool, vomiting, constipation, and finally severe inflammation and swelling of the abdomen. The severity may be mild at first, but as the disease progresses, symptoms can quickly become serious.

Intestinal obstruction can be complete or partial, which restricts the movement of food or stool in the intestine. The chronic obstruction can present with chronic abdominal pain, bloating, vomiting, constipation, and diarrhea [4].

Midgut volvulus is rare in the adults [3, 5]. Our case was an adult. In the adults with malrotation, midgut volvulus is the most common cause of intestinal obstruction. Delay in diagnosis or treatment of midgut volvulus can lead to intestinal necrosis [5].

Irritable bowel syndrome, adhesions, Crohn's disease, pancreatic and biliary disease, and even psychiatric illness are of differential diagnosis that has the same symptoms and this misdiagnosis occurred in the current case for a long time. Therefore, abdominal CT scan is useful and should be considered in the patients with abdominal pain with unknown etiology [3].

On the CT scan, classic whirl pattern around the central superior mesenteric artery is pathognomonic for midgut volvulus that can be helpful in the rapid diagnosis and surgical treatment [6].

Six key steps in the operative correction of malrotation include entry into abdominal cavity and evisceration, counterclockwise distortion of the bowel (acute cases), a division of Ladd's cecal bands, broadening of the small intestine mesentery, incidental appendectomy, and placement of small bowel along with the right lateral gutter and colon along the left lateral gutter. However, Ladd's procedure is most commonly performed [1].

## 5. Conclusion

Midgut volvulus should be suspected in every patient presenting with abrupt onset of pain and sign of intestinal obstruction without obvious causes. The CT scan allows the rapid diagnosis and surgical treatment of the adult patient presenting with abdominal pain or bowel obstruction of uncertain cause. Early diagnosis and immediate operative intervention are key factors associated with a better prognosis for these patients to prevent necrosis and death.
